# Patients with pretreatment leukoencephalopathy and older patients have more cognitive decline after whole brain radiotherapy

**DOI:** 10.1186/s13014-020-01717-x

**Published:** 2020-11-25

**Authors:** Matthew Chan, David Ferguson, Elaine Ni Mhurchu, Ren Yuan, Lovedeep Gondara, Michael McKenzie, Robert Olson, Brian Thiessen, Nafisha Lalani, Roy Ma, Alan Nichol

**Affiliations:** 1 Department of Surgery, Faculty of Medicine, University of British Columbia, Vancouver, BC Canada; 2 Department of Radiation Oncology, BC Cancer - Vancouver, 600 West 10th Ave, Vancouver, BC V5Z 4E6 Canada; 3 Department of Radiology, BC Cancer - Vancouver, Vancouver, BC Canada; 4 Department of Population Oncology, BC Cancer – Vancouver Centre, Vancouver, BC Canada; 5 Department of Radiation Oncology, BC Cancer – Prince George, Prince George, BC Canada; 6 Department of Medical Oncology, BC Cancer – Vancouver, Vancouver, BC Canada

**Keywords:** MRI, White matter hyperintensities, Brain metastases, Radiotherapy, Cognition, Montreal Cognitive Assessment, Fazekas score

## Abstract

**Purpose:**

To investigate predictors of cognitive decline after whole brain radiotherapy (WBRT) for brain metastases.

**Methods:**

A secondary analysis of a phase 2 clinical trial was conducted in patients who received stereotactic radiosurgery for 1–10 brain metastases and WBRT (NCT01046123). The Montreal Cognitive Assessment (MoCA) was performed at baseline and every 3 months after WBRT. Baseline T2-weighted fluid attenuation inversion recovery magnetic resonance imaging was independently assessed by two neuroradiologists for the presence of white matter hyperintensities (WMH) using the Fazekas visual rating scale. WMH were also manually segmented for volumetric analysis. Univariable and multivariable logistic regression were used to test the association between baseline variables and MoCA score decline.

**Results:**

Forty-six patients survived ≥ 3 months after treatment. Age (OR 1.12 (1.04–1.21), *p* < 0.01), baseline WMH volume (OR 1.20, 95% CI 1.06–1.52, *p* = 0.02) and baseline Fazekas score ≥ 3/6 (OR 6.4, 95% CI 1.7–24.7, *p* < 0.01) were predictive of MoCA score decline. In multivariable analysis, age was the only significant predictor of MoCA decline. However, all three patients with pre-treatment leukoencephalopathy (Fazekas score = 6/6) had notable adverse outcomes due to cognitive impairment: one required full-time home nursing support and two were institutionalized.

**Conclusion:**

A greater decline in cognition after WBRT was observed in older patients and patients with a higher baseline WMH burden. Although this study is small and hypothesis-generating, we propose that radiation oncologists should exercise caution in prescribing WBRT if leukoencephalopathy is present on pre-treatment imaging.

*Trial Registration*: clinicaltrials.gov identifier NCT01046123. First posted January 11, 2010. https://clinicaltrials.gov/ct2/show/NCT01046123

## Background

Cognitive impairment after whole brain radiotherapy (WBRT) is a well-documented side effect. Major randomized trials have shown worse neurocognitive function in patients treated with WBRT than in patients treated with SRS, with modern practice shifting towards SRS utilization to treat an increasing number of brain metastases with increasing prescriptions [1–5]. Other randomized trials have shown success in reducing the cognitive impairment from WBRT with memantine and hippocampal avoidance (HA) [6, 7]. The NRG CC001 trial determined that the risk of cognitive failure was significantly lower after HA-WBRT than conventional WBRT. The report included a multivariable Cox proportional hazards model for time-to-cognitive failure which found that younger age (≤ 61 versus > 61) was also a significant predictor of reduced risk of cognitive failure, with a hazard ratio of 0.635 (95% CI 0.479–0.842, *p* = 0.0016). In addition, age was shown to predispose patients to a higher risk of new neurocognitive impairment with 36 Gy, compared to 25 Gy prophylactic cranial irradiation (PCI) in the RTOG 0212 trial [8]. In a multivariable model, age over 60 predicted for a decline in the Hopkins verbal learning test-delayed recall (HVLT) at 12 months in Gondi’s RTOG 0212 and 0214 analysis [9].

The presence of white matter hyperintensities (WMH) in the brain, defined as elevated T2 signal on magnetic resonance imaging (MRI), negatively impacts multiple cognitive domains [10–12]. WBRT is known to cause confluent WMH, or leukoencephalopathy, in many long-term survivors [13]. A small study by Sabsevitz et al. [14] found that pretreatment WMH was a predictor for worse white matter changes after WBRT. The same group also published a secondary analysis of NRG Oncology’s Radiation Therapy Oncology Group 0933 phase 2 clinical trial of HA-WBRT showing that a higher baseline volume of WMH on MRI before radiotherapy (RT), predicted for worse memory decline on HVLT-revised at 4 months [15]. Whether greater WMH on pretreatment imaging increases the risk of global cognitive decline after WBRT remains a question. Confirmation of a relationship between cognitive impairment and the amount of pre-treatment WMH could guide clinicians when deciding between observation, stereotactic radiosurgery (SRS), surgery, and WBRT to manage brain metastases [2, 16, 17].

**Fig. 1:**
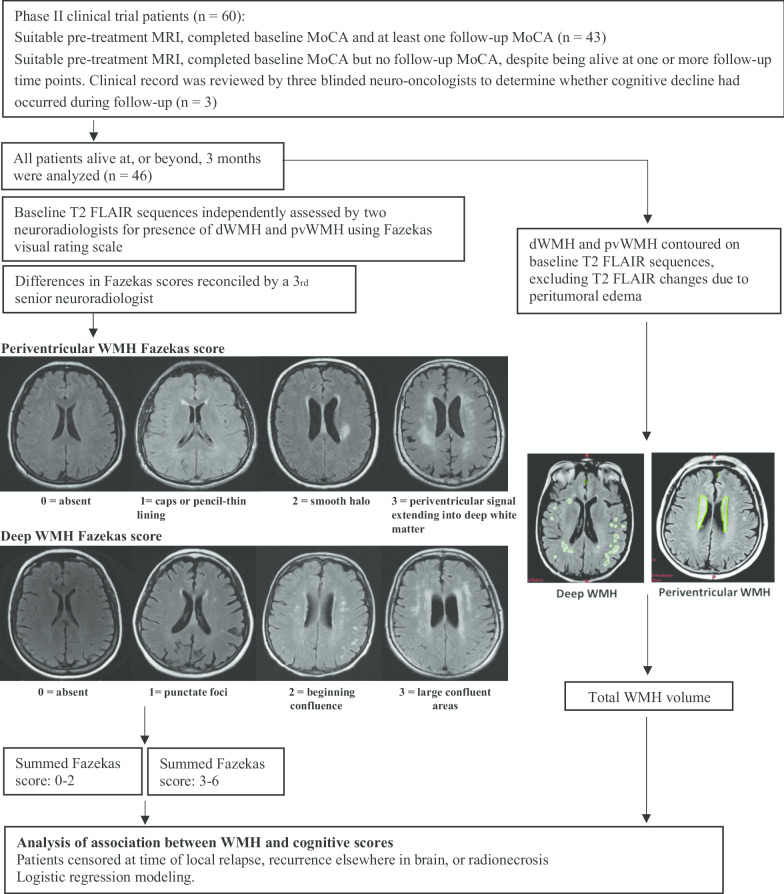
Study methodology. WMH volumes were contoured and scored using the Fazekas visual rating scale on pre-treatment T2-weighted MRI sequences. Changes in MoCA score after whole brain radiotherapy were assessed for associations with age, WMH volume and Fazekas scores. *MoCA* Montreal Cognitive Assessment, *FLAIR* fluid attenuation inversion recovery, *dWMH* deep white matter hyperintensity, *pvWMH* periventricular white matter hyperintensity

WMH can signify multiple pathologic processes including: ischemic lesions, small vessel disease, myelin sheath breakdown, micro-hemorrhage, and disruption of the cerebrospinal fluid-brain barrier [18–20]. The prevalence of WMH in the general population has been shown to increase from 11 to 21% in a cohort with a median age of 64, to 94% in a cohort with a median age of 82 [21, 22]. Hence, it is considered a metric of physiologic brain age [20]. Several methods of quantifying WMH have been described, including the use of visual rating scales and volumetric methods, such as manual contouring and semi-automated intensity-based segmentation [23–25]. Semi-automated methods work well for studies of single pathologies like vascular dementia or multiple sclerosis, but they are confounded by peritumoural edema in patients with brain metastases. Manual volumetric contouring can mitigate the confounding effects of brain metastases, but it is time-consuming and may provide falsely low WMH volumes when there is significant peri-metastatic edema. In contrast, human raters using visual rating scales can disregard metastasis-related edema and measure WMH swiftly. The Fazekas classification is the most widely used rating system for measuring WMH [9]. Fazekas et al. defined periventricular WMH (pvWMH) as being contiguous with the lateral ventricles and deep WMH (dWMH) as being separate from the lateral ventricles. Most studies of WMH have reported cognitive outcomes in relation to the summed (dWMH + pvWMH} Fazekas score (Fig. 1) [26].

The Montreal Cognitive Assessment (MoCA) was developed as a brief screening tool for mild cognitive impairment in geriatric patients [27]. The test items assess eight cognitive domains: orientation, immediate and delayed recall, abstraction, language, visuospatial/executive functioning, attention, naming, and concentration/calculation. Compared to formal neurocognitive testing, a MoCA score of 26–30 has 90% sensitivity and 87% specificity, as well as 89% positive predictive value and 91% negative predictive value for mild cognitive impairment. It has been validated for patients with brain metastases and over a dozen other neurological conditions [28–32]. There are three versions to mitigate practice effects when it is repeated and it has been validated for detecting change in cognition over time [33, 34].

Our group published a phase II trial in 2016 showing similar control and long-term toxicity of 38 Gy in 5 fractions volumetric radiosurgery (using conventional linear accelerators and volumetric modulated arc therapy) compared to published reports of conventional single-fraction radiosurgery [35]. Concurrent 20 Gy in 5 fractions WBRT (EQD2 = 30 Gy/15 for alpha/beta = 2) was delivered with the radiosurgery [36]. MoCA testing was conducted at baseline and used to track the patients' cognition in follow-up. Hypothesizing that patients with higher WMH burden would experience greater declines in their follow-up MoCA test scores after radiotherapy, we conducted a secondary analysis assessing the relationship between baseline WMH and the cognitive side effects of WBRT [29, 35].

## Materials and methods

### Patients

This unplanned secondary analysis was performed with the approval of our research ethics board and all patients provided written consent before entering the phase 2 clinical trial (NCT01046123). Eligible patients were > 18 years with a non-hematologic malignancy. They had 1–10 brain metastases measuring less than 3 cm, Karnofsky Performance Score (KPS) ≥ 70, an estimated median survival of ≥ 6 months, and a baseline MoCA score ≥ 20. Patients with previous craniotomy or brainstem metastases were eligible if they had ≥ 1 unresected, non-brainstem metastasis. There were no patients with upfront leptomeningeal involvement.

### Radiation therapy

A T1-weighted three-dimensional gadolinium-enhanced MRI sequence and a computed tomography (CT) scan with intravenous contrast were performed for radiotherapy planning using a slice reconstruction every 1.00–1.25 mm within a week of the planning CT. The Eclipse treatment planning system (Varian Medical Systems, Palo Alto, CA) was used to co-register the MRI and CT studies, and to segment the metastases and organs at risk. Both the segmented metastasis volumes and whole brain clinical target volumes were expanded by 2 mm to create planning target volumes (PTVs) [37]. The radiosurgery prescription for the brain metastasis PTVs was 38 Gy in 5 fractions. The whole brain PTV was covered by 95% of 20 Gy in 5 fractions. This WBRT prescription was chosen because it could be delivered concurrently with 5-fraction radiosurgery and because it provided equivalent overall survival to 40 Gy in 20 fractions in a randomized clinical trial [38]. Based on the equivalent dose in 2 Gy fractions (EQD2) formulation and assuming an alpha–beta ratio of 10 for brain metastases, we appreciated that 20 Gy in 5 fractions (EQD2 = 23.3 Gy) would offer less cancer cell kill than the more commonly used 30 Gy in 10 fractions (EQD2 = 32.5 Gy); however, the brain metastases were being treated with radiosurgery. We hypothesized that 20 Gy in 5 fractions would provide a similar EQD2 to 25 Gy in 10 fractions (26 Gy) prophylactic cranial radiotherapy and would, therefore, be sufficient to prevent the appearance of new brain metastases. We also wanted to use 20 Gy in 5 fractions because EQD2 calculations indicate that it would cause less late brain toxicity than 30 Gy in 10 fractions: for an alpha/beta ratio of 2, EQD2 = 30 Gy for 20 Gy in 5 fractions versus EQD2 = 37.5 Gy for 30 Gy in 10 fractions. Patients were prescribed dexamethasone 4–16 mg daily during treatment and was either stopped or tapered afterward.

Baseline and follow-up assessments included cognitive testing with MoCA every 3 months until 1 year, and then every 6 months. Practice effects were mitigated by using the three versions of the English MoCA test in rotation. If a patient was still alive 3 months after RT, but failed to complete any follow-up cognitive testing, their attending oncologists were contacted for information about their clinical course and their clinical charts were independently reviewed by three neuro-oncologists to determine whether the patient had experienced a grade 3 cognitive toxicity by the Common Terminology Criteria for Adverse Events (CTCAE) version 4.0 classification [39]. Some factors considered were: severe decline in performance status in the absence of progressing extracranial disease and in the absence of local brain progression or new brain metastases on brain imaging. If the consensus among the neuro-oncologists matched the opinion of the attending oncologists that the patient had grade 3 cognitive toxicity from RT, a cognitive decline event at 3 months was entered in the logistic regression analysis.

### Evaluation of WMH

T2-weighted fluid attenuation inversion recovery magnetic resonance imaging (FLAIR MRI) sequence with a slice thickness of 3–5 mm was used to evaluate WMH. The T2 FLAIR diagnostic imaging was obtained within a month of starting radiotherapy. The neuroradiologists had access to the patients’ health records but not to their cognitive testing results. WMH were independently assessed by two study neuroradiologists using the Fazekas visual rating scale. Disagreements in Fazekas scores between the two study neuroradiologists were resolved by a senior neuroradiologist. For volumetric analysis, M.C. manually segmented the deep and periventricular WMH on the T2 FLAIR sequence in the treatment planning system. The edema associated with brain metastases was also segmented and quantified.

### Statistical analysis

The cognitive outcomes of patients were censored at the time of radionecrosis, local relapse, or untreated recurrences elsewhere in the brain. However, to preserve the study sample size in follow-up, patients remained in the cognitive analysis so long as their MoCA score remained unchanged, or improved, after SRS for new brain metastases. Baseline characteristics were summarized with descriptive statistics. ANOVA eta was used to test association between Fazekas scores and segmented volumes and between Fazekas scores and age. Linear regression was used to investigate the relationship between log transformed WMH Volume and age. Correlations of the pretreatment MoCA score and five baseline variables were calculated: (1) age, (2) WMH volume, (3) Fazekas score, (4) number of metastases, and (5) volume of peritumoural edema. Logistic regression was used to determine the relationship between baseline variables and the coded MoCA score (0 = stable or improved, 1 = declined). The summed Fazekas score was categorized into two groups with scores 0–2 and 3–6 [40, 41]. The WMH volume used in the analyses excluded the peritumoral edema and was defined as the sum of the volume of periventricular WMH and deep WMH. Age and WMH volume were analysed as continuous variables. All tests were two-sided, with *p* < 0.05 considered statistically significant. The data were analyzed using SPSS version 21.0 (IBM, Armonk, NY) and R [42].

## Results

### Participant and baseline characteristics

Patients’ baseline characteristics are shown in Table 1. Forty-six patients were analyzed: forty-three who had cognitive testing after RT and three who did not, but were alive for at least 3 months after WBRT. The median age was 60.5 years (range: 35–82) and the median MoCA score was 27.5. Patients had a median of 3 brain metastases causing a mean volume of 38.4 cm^3^ of peritumoral edema.

**Table 1:** Patient, tumor, and treatment characteristics

Variable	All	Fazekas score			
	Patients	0–2	3–6	0–5	6
% patients (n)	100% (45)	57% (26)	43% (20)	93% (43)	7% (3)
Age (years)					
Median	60.5	54.5	65.5	59.0	72.0
Range	35–82	35–74	46–82	35–82	70–82
≤ 65	71.7%	88%	50%	77%	0%
Baseline KPS					
Median	85	90	80	80	80
70–80	50%	44%	65%	51%	100%
90–100	50%	56%	35%	49%	0%
Highest education^a^					
Median	13	13	13	13	13
Range	11–13	11–13	11–13	11–13	12–13
Baseline MoCA					
Median	27.5	27.5	27.5	28	26.0
Range	20–30	20–30	20–30	20–30	20–30
Metastases (no.)					
Median	3	3.5	2.5	3	1
Range	1–10	1–10	1–10	1–10	1–3
Volume of metastases (cm^3^)					
Mean	4.1	5.0	3.0	4.0	5.2
Peritumoral edema^b^ (cm^3^)					
Mean	38.4	38.1	38.9	38.4	39.7
Intracranial volume (cm^3^)					
Mean	1531	1524	1542	1530	1557
WMH volume (cm^3^)					
Mean	8.0	1.8	16.0	5.0	50.4

*KPS* Karnofsky performance status, *MoCA* Montreal Cognitive Assessment

^a^13 = any post-secondary education

^b^Excludes volume of metastases

### Baseline variables

**Fig. 2:**
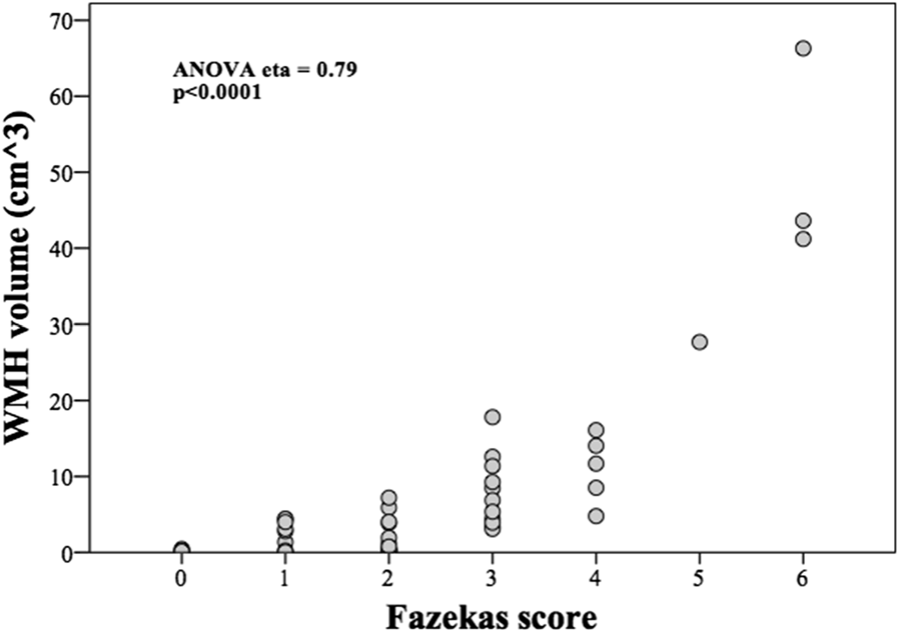
Correlation between segmented WMH volume and Fazekas score. A strong and statistically significant association was seen between Fazekas scores and segmented WMH volumes. *WMH* white matter hyperintensity

There were only 6/46 pvWMH and 8/46 dWMH disagreements between neuroradiologists in initial Fazekas scoring, all by only one point. This occurred most frequently between Fazekas scores of 1 versus 2 (4/6 pvWMH and 5/8 dWMH). Strong correlations were seen between Fazekas scores and segmented volumes for pvWMH (eta = 0.91, *p* < 0.001) and dWMH (eta = 0.91, *p* < 0.001), as well as between the Fazekas scores and total segmented volumes (eta = 0.79, *p* < 0.0001) (Fig. 2). WMH volume was not normally distributed, so to make linear regression possible, it was transformed with Log (WMH volume + 1). There was a strong linear relationship between age and Log (WMH volume + 1): (0.025 * age − 0.82, *p* < 0.0001). Correlation was also observed between Fazekas score and age (eta = 0.58, *p* = 0.009).

### Cognition at baseline

Correlations of the pretreatment MoCA scores and five baseline variables were calculated. There were no significant relationships between baseline MoCA score and age: − 0.15 (*p* = 0.16), number of metastases: 0.10 (*p* = 0.36), volume of peritumoral edema: − 0.20 (*p* = 0.06), WMH volume: − 0.15 (*p* = 0.16) or Fazekas score: (0.27) *p* = 0.78.

### Changes in MoCA scores

**Fig. 3:**
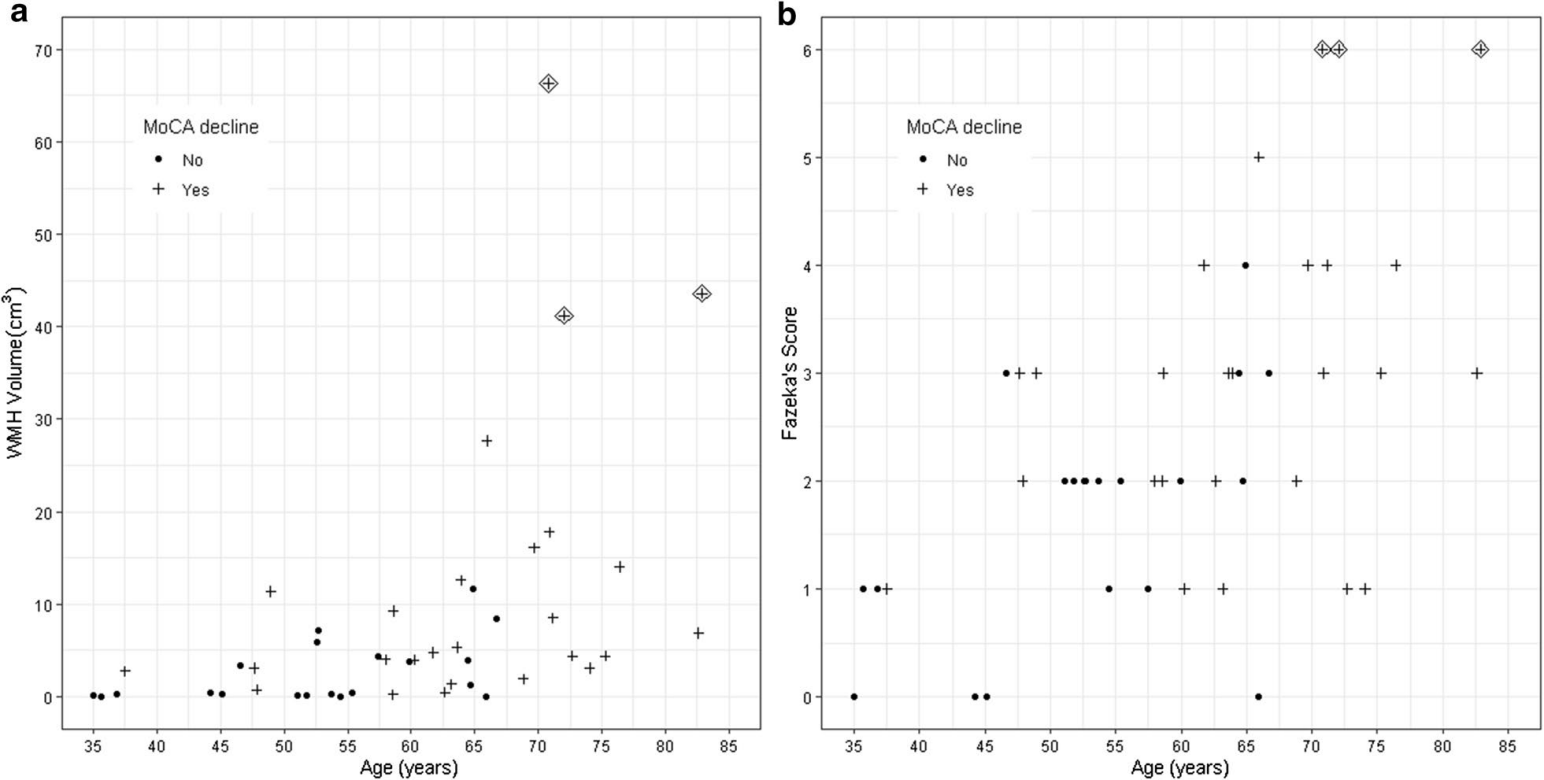
Changes in MoCA score after whole brain radiotherapy. Plotted with age and **a** pretreatment WMH volume and **b** pretreatment Fazekas score. The diamond-shaped data points are those of patients with a baseline Fazekas score of 6. *WMH* white matter hyperintensity, *MoCA* Montreal Cognitive Assessment

Associations between WMH burden and decline from baseline MoCA score were assessed by logistic regression (Table 2). In three univariable logistic regression models, increasing age (OR 1.12 (1.04–1.21), *p* = 0.003), increasing baseline WMH volume (OR 1.20, 95% CI 1.06–1.52, *p* = 0.02) and baseline Fazekas score 3–6 versus 0–2 (OR 6.4, 95% CI 1.7–24.7, *p* = 0.007) were predictive of MoCA score decline. Eighty percent (16/20) of patients with Fazekas Score 3–6 had cognitive decline and 50% (13/26) of patients with Fazekas score 0–2 had cognitive decline. All ten patients over the age of 70 experienced cognitive decline. In two bivariable logistic regression models, of age with WMH volume and age with Fazekas score, age was the only significant predictor of MoCA decline. The graphical relationships between WMH volume, MoCA decline and age and between Fazekas score, MoCA decline and age are illustrated in Fig. 3. Patients with a Fazekas score of 0–2 had a median MoCA score decline of 0.0, while patients with a Fazekas score of 3–6 had a median MoCA score decline of 3.0 after WBRT.

**Table 2:** Logistic regression analyses of changes in MoCA score from baseline after WBRT

	∆ MoCA score: baseline versus worst follow-up score		Odds ratio (95% CI) for MoCA score decline	*p* value
	Median	Mean		
Univariate analyses				
Age^a^			1.12 (1.04–1.21)	0.003
WMH volume^a^			1.20 (1.06–1.52)	0.02
Fazekas score (0–2); (Odds ratio: 0–2 versus 3–6)	0.0	− 1.7	6.4 (1.7–24.7)	0.007
(3–6)	− 3.0	− 3.5		
Number of metastases (1–10)			1.0 (0.8–1.2)	1.0
Volume of edema			1.005 (0.99–1.02)	0.4
Bivariate analyses				
Age^a^			1.08 (0.99–1.16)	0.05
and WMH volume^a^			1.14 (0.96–1.37)	0.14
Age^a^			1.09 (1.01–1.18)	0.02
and Fazekas score (0–2 versus 3–6)			3.1 (0.7–14.1)	0.1

*MoCA* Montreal Cognitive Assessment, *WMH* white matter hyperintensity

^a^Continuous variables

### Neuro-oncologist review

There were three patients who were alive for at least 3 months after RT but did not have any follow-up MoCA score results. Three neuro-oncologists reviewed their medical records for clinical evidence of cognitive decline. At 3 months, one patient had normal cognition documented by their attending oncologist in the context of rapidly progressing extracranial disease. The neuro-oncologists agreed that no cognitive decline event had occurred in this patient. However, they agreed that a cognitive decline event had occurred in the other two patients. At 3 months, these two patients did not have extracranial or intracranial disease progression or social factors that explained their failure to complete cognitive assessments. One was admitted to a nursing home because of confusion and the other was admitted to a hospice with somnolence syndrome. These two patients with severe clinical cognitive decline (equivalent to CTCAE grade 3 cognitive toxicity) both had baseline Fazekas scores of 6. All three patients with Fazekas scores of 6 had cognitive decline that was severe enough to be scored as CTCAE grade 3 toxicity (Table 3).

**Table 3:** Clinical histories of all three patients with Fazekas scores of 6

Patient number	Age	WMH volume (cm^3^)	∆ MoCA score: baseline versus worst follow-up score	Outcome
1	72	41.2	8-Point decrease at 6 months	Somnolence for several months after WBRT with short-term memory and concentration impairment requiring full-time nursing support. An MRI 6 months after treatment showed control of brain metastases and worsening leukoencephalopathy. Lived for 18 months after WBRT
2	83	43.6	Did not complete a follow-up MoCA test	Admitted to nursing home with confusion 2 months after WBRT. Did not attend any follow-up clinical trial appointments. An MRI 1 year after treatment showed control of brain metastases and new leukoencephalopathy. Lived for 28 months after WBRT in a nursing home
3	71	66.3	Did not complete a follow-up MoCA test	Three-month MRI showed control of brain metastases, but she did not attend the 3-month clinical trial appointment because she was sleeping 23 h per day, compatible with somnolence syndrome. Lived for 6 months after WBRT in a hospice

*WMH* white matter hyperintensity, *WBRT* whole brain radiotherapy, *RT* radiotherapy, *MoCA* Montreal Cognitive Assessment

## Discussion

Our use of the Fazekas visual rating scale to assess WMH before RT is novel. The simplicity and speed of the Fazekas rating scale would make its use by radiation oncologists more practical than volumetric segmentation in a clinic setting when trying to decide whether WBRT would be appropriate for a given patient [27, 28]. This study found that age (*p* = 0.003), WMH volume (*p* = 0.02) and Fazekas score (*p* = 0.007) are all significant predictors of cognitive decline after WBRT. In multivariable analyses age remained the only significant predictor of cognitive decline in the model with Fazekas score. Hence, our results confirm the findings of Brown et al*.* that older age was an important predictor of cognitive decline in their multivariable analysis of the CC001 study. It is noteworthy that every patient over 70 in our study experienced cognitive decline after WBRT.

To our knowledge, this the second study to demonstrate that the volume of pre-treatment WMH is a risk factor for cognitive decline after WBRT. WMH is an MRI biomarker for age-related microvascular injury in the brain, so we were not surprised to find that age had a significant linear relationship with log transformed WMH volume (*p* < 0.0001) and Fazekas score (*p* < 0.0001). We also found that our two metrics of WMH burden in the brain: WMH volume and Fazekas score, were highly correlated. Our volumetric WMH findings substantiate those from a secondary analysis of a subset of 33 patients treated with hippocampal the RTOG 0933 clinical trial. As we did, Bovi et al. found that a significant correlation between greater baseline WMH volume and older age (rho = 0.38, *p* = 0.03) could be detected in patients with brain metastases despite the presence of potentially confounding peritumoural edema. They also found that the change in the Hopkins Verbal Learning Test—Revised immediate recognition memory at 4 months after WBRT was correlated with baseline WMH volume (rho = 0.54, *p* = 0.001), consistent with a greater burden of WMH being associated with a greater decline in a single subtest of memory. In contrast, we used the MoCA test, which assesses eight neurocognitive domains, to study change in cognition. We believe that use of a global cognitive measure increases the robustness of our finding that WMH burden predicts cognitive decline after RT.

A counter-intuitive strength of this study is that it was performed at a single institution: we had full access to the patients’ clinical records, follow-up imaging and attending oncologists. As opposed to a multi-institutional study, in which analyses can only be performed with the data collected on case-report forms and case-report forms can only be completed for patients who attend appointments, we could find out what happened to patients who would have otherwise have been lost to follow-up. It is clear from Fig. 3 that older patients have higher WMH volumes and Fazekas scores than younger patients. However, this plot also shows that there is a subset of older patients with very high WMH volumes and high Fazekas scores. Our study showed that this subset of patients have particularly bad clinical outcomes. After WBRT, the three patients with a Fazekas Score of 6 lived, respectively, for 18 months with full-time home care nursing, 28 months in a nursing home and 6 months in a hospice. Our observation that a Fazekas score of 6 was associated with profound cognitive impairment after WBRT is based on only three patients. Yet, we feel it is important to alert the radiation oncology community about this observation because it is biologically plausible that WBRT could cause precipitous cognitive impairment in patients with extensive microvascular damage to the brain and minimal cognitive reserve. It should also be noted that while studies have suggested that patients with underlying demyelinating disease (e.g. multiple sclerosis) are at risk for severe neurotoxicity after brain radiotherapy, no patients in our cohort had clinical histories or imaging consistent such pathology [43]. Based on our results, we suggest that a Fazekas score of 6 should be considered a relative contraindication to WBRT and that alternatives such as surgery, SRS, systemic therapy, or observation should be strongly considered. The randomized QUARTZ trial showed no difference in quality of life between WBRT and best supportive care in unselected lung cancer patients who were ineligible for surgery or SRS [16]. However, our findings suggest that for patients with elevated Fazekas scores, WBRT may cause worse clinical outcomes than observation. Our findings also suggest that clinicians should also assess the WMH burden on pretreatment MRI when considering prophylactic cranial irradiation for small cell lung cancer. We hope that our report will prompt more research in this area.

There are advantages and disadvantages to the two methods we used to measure WMH burden. Segmentation can achieve a more rigorous, quantitative determination of WMH burden; however, due to a lack of well-defined contouring guidelines in the literature, there was uncertainty in some instances. Also, segmentation underestimates the age-related WMH burden when extensive peri-metastatic edema obscures age-related WMHs. According to our three neuro-radiologists, the use of the Fazekas visual rating scale overcame these contouring uncertainties by allowing them to “see past” the generally asymmetric peri-tumoural edema and focus instead on the distribution of contralateral age-related WMH. In Fazekas scoring, there was difficulty in differentiating a normal, thin periventricular increase in T2 signal (pvWMH Fazekas = 0) from a thin, but abnormal, increase in T2 signal along the lateral ventricle (pvWMH Fazekas = 1), due to variation in FLAIR-sequence intensity and slice thicknesses between scans [44, 45]. It was also sometimes difficult to distinguish an irregular periventricular WMH cap at the end of the lateral ventricle from confluent punctate dWMH in the parenchyma. These challenges in rating low volumes of WMH led us to consider Fazekas score = 0–2 as a “no to radiologically questionable” WMH category, distinct from Fazekas score = 3–6, which we considered as a “radiologically certain” WMH category.

Using the Fazekas score, Mayinger et al. measured the development of post-treatment WMH in patients receiving WBRT and hippocampal-avoidance WBRT (HA-WBRT) for prophylactic cranial irradiation [46]. The nine patients in each cohort had the same Fazekas score at baseline, but they found that, while the median Fazekas score was 2 after HA-WBRT, it was only 1 after conventional WBRT. They also observed that there was a significant Fazekas score increase over time after HA-WBRT (*p* = 0.001). They hypothesized that dose inhomogeneity in the HA-WBRT plans, which had a higher Dmax and greater volumes receiving more than the prescription dose might be responsible for these differences. In our study, patients received WBRT and SRS, so there were small volumes of normal brain that received a high dose and more dose inhomogeneity in our patient’s plans than in conventional WBRT plans. However, in 1-year survivors after radiotherapy for brain metastases, Monaco et al. showed that only 3% (1/33) developed leukoencephalopathy after SRS compared to 97.7% (36/37) after WBRT. With this in mind, we believe that the small volumes of brain exposed to SRS doses in our study would have had little influence our main study endpoint. Had this been influential, its effect would have been to hasten and worsen cognitive decline in patients with more and larger brain metastases, but we found no association between number of metastases and MoCA decline.

Our study must be interpreted within the context of its strengths and limitations. Due to our small sample size, we could not control for potential confounders such as education level, number of brain metastases and intracranial volume (as a surrogate for brain volume and cognitive reserve). Also, we could not correlate changes in WMH or edema after radiotherapy with MoCA scoring because the patients enrolled in our phase II clinical trial were followed for local progression and new brain metastases with only gadolinium-enhanced T1 sequences. Although we accounted for local progression, radionecrosis, and new brain metastases by censoring patients with these events, we did not measure metastases after WBRT. Hence, we may have missed some cognitive decline events from progressive WMH that were masked by improving cognition from shrinking metastases and peri-metastatic edema. An analysis with a larger cohort should investigate whether the overall radiological response of the brain metastases is associated with improved cognition within cohorts of patients of matched baseline WMH burden. Our use of 20 Gy in 5 fractions may limit the generalizability of our findings to centres that use 30 Gy in 10 fractions [36, 47, 48]. Unfortunately, we did not acquire information during follow-up about systemic therapies that may have affected cognition. In our phase II clinical trial, using the MoCA was a practical decision based on the need to test cognition in 10 min during brief follow-up appointments. Although it tests eight cognitive domains, it may have missed subtle cognitive decline that lengthier neurocognitive testing would have detected, causing our study to underestimate the effects of baseline WMH on cognitive decline. A larger sample size with more detailed neurocognitive testing would allow us to confirm our findings. We have secured funding to perform a secondary analysis of baseline imaging and post-WBRT cognition in two large phase 3 trials that included neurocognitive testing [2, 3].

## Conclusion

We observed diminished cognitive function after whole brain radiotherapy in older patients and especially poor clinical outcomes in older patients with pre-treatment leukoencephalopathy. Although our study is small and hypothesis-generating, we urge radiation oncologists to review pre-treatment imaging for leukoencephalopathy and, if present, consider treatment options other than WBRT.

## Data Availability

The datasets used and/or analysed during the current study are available from the corresponding author on reasonable request.

## Abbreviations

WBRT: Whole brain radiotherapy
MoCA: Montreal Cognitive Assessment
WMH: White matter hyperintensities
SRS: Stereotactic radiosurgery
HA: Hippocampal avoidance
PCI: Prophylactic cranial irradiation
HVLT: Hopkin’s verbal learning test
MRI: Magnetic resonance imaging
RT: Radiotherapy
pvWMH: Periventricular white matter hyperintensities
dWMH: Deep white matter hyperintensities
KPS: Karnofsky Performance Score
CT: Computed tomography
PTV: Planning target volume
FLAIR: Fluid attenuation inversion recovery
